# COVID-19-specific risk factor for early post-appendectomy complications (EPAC) in older patients: a retrospective study

**DOI:** 10.1007/s10151-025-03232-1

**Published:** 2025-11-05

**Authors:** Tamer A. A. M. Habeeb, A. Hussain, Jose Bueno-Lledó, M. E. Giménez, A. Aiolfi, M. Chiaretti, I. A. Kryvoruchko, M. N. Manangi, Abd Al-Kareem Elias, Abdelmonem A.M Adam, Mohamed A. Gadallah, Saad Mohamed Ali Ahmed, Ahmed Khyrallh, Mohammed H. Alsayed, Esmail Tharwat Kamel Awad, Emad A. Ibrahim, Mohammed Hassan Elshafey, Mohamed fathy Labib, Mahmoud Hassib Morsi Badawy, Sobhy rezk ahmed Teama, Abdelhafez Seleem, Mohamed Ibrahim Abo Alsaad, Abouelatta KH Ali, Hamdi Elbelkasi, Mahmoud Ali abou zaid, Basma Ahmed Mohamed, Alaa Alwadees, Ahmed k. El-Taher, Mohamed Ibrahim Mansour, Mahmoud Abdou Yassin, Ahmed Salah Arafa, Mohamed Lotfy, Baher Atef, Mohamed Elnemr, Mostafa M Khairy, Abdelfatah H. Abdelwanis, ahmed mesbah Abdelaziz, Abdelshafy Mostafa, AbdElwahab M. Hamed, Tamer Wasefy, Ibrahim A. Heggy, Abdelrahman Mohamed Hasanin Nawar

**Affiliations:** 1 Department of General Surgery, Department of General Surgery, Faculty of Medicine, Zagazig University, Zagazig, Egypt; 2 Sheffield University, Sheffield, UK; 3 University of Alkafeel, Najaf, Iraq; 4 Hospital Universitari i Politècnic la Fe, Valencia, Spain; 5 Image-Guided Surgery, IHU-IRCAD, University of Strasbourg, Strasbourg, France; 6 Division of General Surgery, Department of Biomedical Science for Health, I.R.C.C.S. Ospedale Galeazzi-Sant’Ambrogio, University of Milan, Milan, Italy; 7 Department of General Surgery Specialties and Organ Transplant, Faculty of Pharmacy and Medicine, Sapienza Rome University, Rome, Italy; 8 Department of Surgery No. 2, Kharkiv National Medical University, Kharkiv, Ukraine; 9 Department of General Surgery, Victoria Hospital, Bengaluru, India; 10 Department of General Surgery, Faculty of Medicine, Al-Azhar University, Assuit Branch, Assuit, Egypt; 11 General Surgery Department, Faculty of Medicine, Al-Azhar University, Cairo, Egypt; 12 General Surgery Department, Faculty of Medicine, Merit University, Sohag, Egypt; 13 Misr University for Science and Technology, Cairo, Egypt; 14 Mataria Teaching Hospital (GOTHI), Cairo, Egypt; 15 General Surgery Department, El Mahala Hepatic Institute, Al Gharbia, Egypt; 16 Department of General Surgery, Faculty of Medicine for Girls, Cairo Al Azhar University, Cairo, Egypt; 17 Jabir Ibn Hayyan University, Najaf, Iraq

**Keywords:** Acute appendicitis, Appendectomy, COVID-19, Older patients, Postoperative complications

## Abstract

**Background:**

The incidence of acute appendicitis in older patients significantly varies from that in younger adults. The coronavirus disease 2019 (COVID-19) pandemic has increased the risk of early post-appendectomy complications (EPAC). This study aimed to investigate the incidence and risk factors associated with EPAC in older patients after appendectomy and to define active COVID-19 infection during surgery as an associated risk factor for EPAC.

**Methods:**

We conducted a retrospective multicenter analysis of older patients aged ≥ 60 years who underwent appendectomy between April 2020 and December 2024. Logistic regression identified the risk factors associated with EPAC.

**Results:**

A total of 585 patients aged ≥ 60 years were divided into the EPAC (*n* = 32) and no EPAC (*n* = 553) groups. The incidences of EPAC was 5.5% (32/585), including superficial incisional surgical site infections (SSI) (9/32, 28.1%), deep incisional SSI (2/32, 6.3%), organ/space infection (2/32, 6.3%), intra-abdominal abscess (9/32, 28.1%), ileus (2/32, 6.3%), pneumonia (3/32, 9.4%), acute myocardial infraction (MI) (2/32, 6.3%), fecal fistula (2/32, 6.3%), and acute adhesive intestinal obstruction (1/32, 3.1%). Multivariable analysis identified that active COVID-19 infection during surgery (odds ratio (OR) = 25.9; 95% confidence interval (CI) 4.8–139.1; *p* < 0.001), American Society of Anesthesiologists (ASA) score ≥ II (OR = 4.5; 95% CI 1.2–17.07; *p* = 0.02), open approach (OR = 30.6; 95% CI 8.1–115.3; *p* < 0.001), and high-grade appendicitis ≥ IV (OR = 63.06; 95% CI 7.5–526.4; *p* < 0.001) were significant associated risk factors for EPAC.

**Conclusions:**

The incidence of EPAC in older patients after appendectomy is 5.5%. Active COVID-19 infection during surgery is strongly associated with an increased risk of EPAC. COVID-19 should be considered in perioperative risk assessment of EPAC.

**Trial registration:**

This study was registered as a clinical trial (NCT06787573). Retrospectively registered.

**Graphical abstract:**

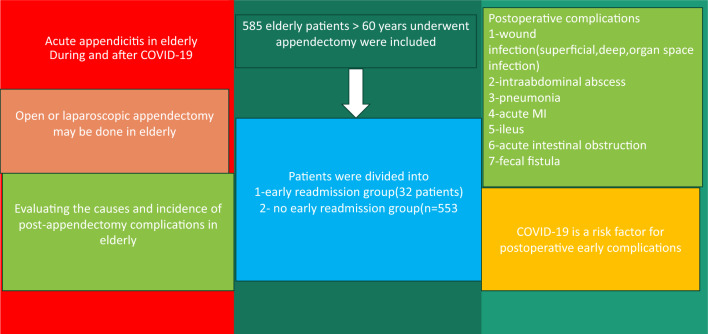

**Supplementary Information:**

The online version contains supplementary material available at 10.1007/s10151-025-03232-1.

## Introduction

Acute appendicitis (AA) is the most common emergency abdominal surgery performed worldwide. Global population growth, increasing life expectancy, and advancements in diagnostic and imaging technologies are anticipated to consistently increase older patients in the coming years [1].

AA is a significant challenge in older patients. These patients often present with atypical symptoms, leading to diagnostic delays in up to 30–35% of patients. Delayed diagnosis increases the risk of complications, including perforation, gangrene, abscess formation, and peritonitis. Moreover, older patients are typically frail, making them unsuitable candidates for emergency surgery, which frequently includes complicated operations and increases postoperative morbidity and mortality [2]. The incidence of severe AA is higher in older patients, with perforation rates approaching 70% and morbidity rates reaching 48% [3]. The most common early post-appendectomy complications (EPAC) include surgical site infections (SSI), intra-abdominal abscesses (IAA), and ileus [4, 5].

The COVID-19 pandemic has prompted the revision of healthcare strategies to mitigate viral transmission. Guidelines from major surgical societies emphasize the safety of patients and personnel, which often alters standard care pathways [6]. Regarding AA during the COVID-19 pandemic, this sometimes led to a preference for open appendectomy (OA) over laparoscopic appendectomy (LA) owing to concerns about viral aerosolization during pneumoperitoneum [4].

Although several studies have examined AA in older patients [7–9], few have specifically evaluated the full spectrum of EPAC within the COVID-19 pandemic. To the best of our knowledge, there are no data in the literature evaluating the incidence and risk factors associated with EPAC within 30 days after appendectomy in older patients during and after the COVID-19 pandemic, and evaluating active COVID-19 infection during surgery as an associated risk factor.

## Methods

### Study design and settings

This was a retrospective multicenter study involving older patients in four tertiary centers in Egypt between April 2020 and December 2024. This study was designed according to the Declaration of Helsinki guidelines and the strengthening of the Reporting of Cohort, Cross-sectional, and Case–Control Studies in Surgery (STROCSS) statement [10]. This study was approved by the Ethics Review Committee of Zagazig University (ZU-IRB#10271). Preoperative consent was provided by the patients for the intervention.

### Eligibility criteria

Inclusion criteria included all consecutive older patients aged ≥ 60 years [https://www.who.int/news-room/fact-sheets/detail/ageing-and-health]; identified using hospital surgical registries and operative logs; diagnosed with AA on the basis of clinical diagnosis, radiological diagnosis, operative findings; who underwent appendectomy (open appendectomy (OA) and laparoscopic appendectomy (LA)); and in whom the diagnosis of AA was confirmed by postoperative histopathology in all patients. Exclusion criteria were patients with acute appendicitis who underwent conservative treatment, cecal diverticulitis, appendectomy for malignant disease of the cecum or appendix, appendectomy during elective surgery, patients with negative acute appendicitis at histologic examination, cognitively impaired, and lost to follow-up.

Site recruitment and volumes: This multicenter study was conducted across four hospitals in Egypt. We included two major university hospitals, Zagazig University and Al-Azhar University, which are known for handling complex cases referred from wider regions. These academic centers see a high volume of patients, performing around 1500 emergency appendectomies each year, including many frail older patients with other health conditions. They benefit from advanced technology, specialist teams, and resident training programs, which shape care delivery. The other two hospitals were Mataryia Teaching Hospital and El Mahala Institute. Rooted in their communities, each handles an estimated 380 emergency appendectomies annually, representing essential frontline surgical care.

### Variables and data collection

1. Demographic and clinical characteristics: age, sex, previous appendicular abscess drainage, American Society of Anesthesiologists (ASA) score, smoking status, white blood cell (WBC) count, coronary heart disease (CHD) COVID-19 infection (active COVID-19 infection during hospital admission, past history of COVID-19 during hospital admission), diabetes mellitus (DM), hypertension (HTN), clinical frailty scale (CFS) [11], age-adjusted Charlson Comorbidity Index (CCI) [12], previous abdominal surgeries, and previous history of episodes of acute appendicitis defined as any previous documented episode of clinically and/or radiologically diagnosed appendicitis managed nonoperatively (antibiotics ± observation) occurring before the index admission during the study period. The CFS is a clinician’s assessment tool that summarizes an older patient’s overall health status before illness. It rates frailty status on a spectrum from “managing well” to “severely frail.” The CCI is a well-established system that predicts mortality and adverse outcomes by measuring a patient’s burden of comorbid conditions. Each condition was assigned a weighted score based on its impact on mortality risk, and the total CCI score was the sum of these weights; higher scores reflected greater comorbidity and associated risk. Acute appendicitis (AA) was diagnosed according to established guidelines [7], with pelvic–abdominal CT used in uncertain cases to exclude other pathologies, particularly malignancies [4]. Localized appendicular abscesses size was defined by the largest diameter in centimetres on CT (< 3 cm and > 3 cm) and managed with radiological drainage and antibiotics. Surgical drainage was performed for failed radiological drainage or when an appendicular abscess was undiagnosed preoperatively, in line with the institution’s protocol. All patients with successful drainage were presented for interval appendectomy.
2. Intraoperative data included surgical approach (LA and OA), duration of surgery (min), operative findings (including laparoscopic grading of appendicitis) [13], and grading of appendicitis during open approach were based on the surgeon’s operative notes, intraoperative complications, and their management.
3. Postoperative data: hospital stay (days), EPAC [including SSI, intra-abdominal abscess, ileus, pneumonia, acute myocardial infarction, fecal fistula and acute adhesive intestinal obstruction], management plan, and mortality rate. The Clavien–Dindo (CD) classification was used to grade postoperative complications [14].

### Definition of outcomes and measurement

The primary outcomes were the incidence and risk factors associated with EPAC within 30 days of surgery. In addition, we aimed to evaluate the association between active COVID-19 infection and the risk of developing EPAC. Our study evaluates the complete range of early postoperative complications defined under EPAC. We adopted the Centers for Disease Control (CDC) classification system for SSI as superficial, deep, and organ/space [15]. Postoperative intra-abdominal abscess (IAA) is characterized by intra-abdominal fluid collection identified via radiologic assessment, accompanied by systemic or localized symptoms [16].

Perioperative protocols and postoperative follow-up are summarized in Supplementary Table 1.

### Statistical analysis

Data management and analyses were performed using SPSS version 28 (IBM Corp., Armonk, New York, United States). Continuous data are presented as mean ± standard deviation or median with interquartile range, whereas categorical data are presented as numbers and percentages. We used the Student’s *t*-test for parametric data or the Mann–Whitney *U* test for nonparametric comparisons. Categorical variables were analyzed using the *χ*^2^ test or Fisher’s exact test. Univariable analyses were conducted for all candidate variables, including COVID-19 status. Variables with a *p* value < 0.25 on univariable analysis were entered into a multivariable logistic regression model to determine associated predictors of EPAC, with results reported as odds ratios (ORs) and 95% confidence intervals (CIs). No data were missing for variables included in the final regression model. This analysis identified statistical associations rather than causal relationships. Statistical significance was set at *p* < 0.05.

## Results

**Fig. 1:**
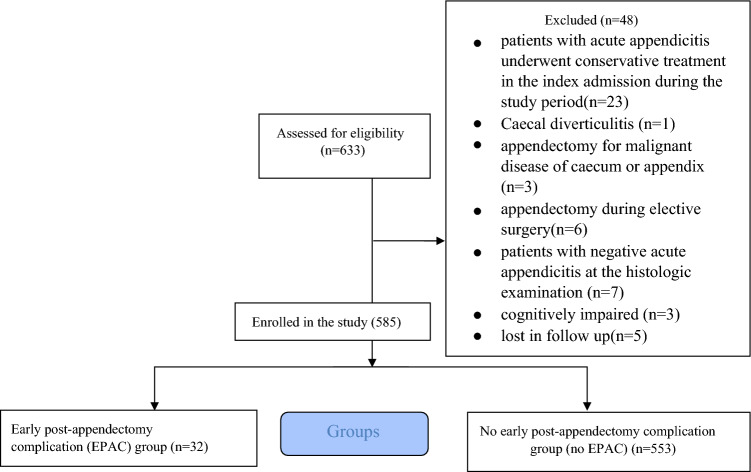
Flowchart of the patient selection process

A total of 585 older patients who underwent appendectomy (OA and LA) were included in the final analysis. The flowchart of the patient selection process is detailed in Fig. 1. Patients were divided into an EPAC group (*n* = 32) and a no EPAC group (*n* = 553).

The demographic and clinical characteristics of patients are presented in Table 1. Compared with the no EPAC group, patients in the EPAC group had significantly higher ASA scores of ≥ II (81.3% versus 29.7%, *p* < 0.001), active COVID-19 infection (77.8% versus 22.2%, *p* < 0.001), DM (59.4% versus 16.1%, *p* < 0.001), HTN (56.3% versus 14.3%, *p* < 0.001), previous history of episodes of acute appendicitis before the index admission (18.8% versus 2.5%, *p* < 0.001), and a significantly lower mean BMI (28.06 ± 4.07 versus 29.69 ± 5.14, *p* = 0.03).

**Table 1:** Demographic and clinical characteristics of the study groups

	Early postoperative complications (EPAC group)	No early postoperative complications (no EPAC group)	*p*-Value
	(*n* = 32)	(*n* = 553)	
Sex			
Male	17 (53.1%)	321 (58%)	0.5
Female	15 (46.9%)	232 (42%)	
Age	66.34 ± 3.8	65.45 ± 3.8	0.2
Previous appendicular abscess drainage	0 (0.00%)	20 (3.6%)	0.2
Size of previous appendicular abscess			
< 3 cm	0 (0.00%)	7 (1.3%)	0.5
> 3 cm	0 (0.00%)	13 (2.4%)	
Type of drainage of the previous appendicular abscess			
Percutaneous sonar-guided drainage	0 (0.00%)	7 (1.3%)	0.7
Percutaneous CT-guided drainage	0 (0.00%)	4 (0.7%)	
Open drainage	0 (0.00%)	9 (1.6%)	
Smoking	5 (15.6%)	101 (18.3%)	0.7
ASA			
ASA-I	6 (18.8%)	389 (70.3%)	< 0.001*
ASA-II	14 (43.8%)	132 (23.9%)	
ASA-III	12 (37.5%)	32 (5.8%)	
WBCS	15.95 ± 0.75	15.75 ± 0.7	0.1
CHD	8 (25%)	90 (16.3%)	0.1
COVID-19 infection			
	17 (53.1%)	77 (13.9%)	< 0.001*
Active COVID-19 infection during hospital admission (*n* = 18)	14/18 (77.8%)	4 (22.2%)	
Past history of COVID-19 during hospital admission (*n* = 76)	3 (4%)	73 (96%)	
DM	19 (59.4%)	89 (16.1%)	< 0.001*
BMI	28.06 ± 4.07	29.69 ± 5.14	0.03*
HTN	18 (56.3%)	79 (14.3%)	< 0.001*
Frailty status			
Managing well	2 (6.3%)	77 (13.9%)	0.6
Well	2 (6.3%)	34 (6.1%)	
Vulnerable	4 (12.5%)	110 (19.9%)	
Mild frail	14 (43.7%)	190 (34.4%)	
Moderately frail	5 (15.6%)	80 (14.5%)	
Severely frail	5 (15.6%)	62 (11.2%)	
Age-adjusted Charlson Comorbidity Index score			
4	2 (6.3%)	23 (4.2%)	0.5
5	14 (43.7%)	164 (29.7%)	
6	4 (12.5%)	143 (25.9%)	
7	5 (15.6%)	70 (12.7%)	
8	3 (9.4%)	65 (11.8%)	
9	3 (9.4%)	43 (7.8%)	
10	0 (0.00%)	4 (0.7%)	
11	1 (3.1%)	41 (7.4%)	
Previous abdominal surgeries	2 (6.3%)	43 (7.8%)	0.7
Previous history of episodes of acute appendicitis before the index admission	6 (18.8%)	14 (2.5%)	< 0.001*

*ASA* American Society of Anesthesiologists, *CHD* coronary heart disease, *DM* diabetes mellitus, *BMI* body mass index, *HTN* hypertension

*Statistically significant

The intraoperative data are summarized in Table 2. Laparoscopic appendectomy (LA) was associated with lower EPAC rates (3.9% versus 7.9% for open appendectomy; *p* = 0.03). The EPAC group showed a significantly higher rate of laparoscopic grading of AA severity of ≥ IV (37.5% versus 7.6%, *p* < 0.001), intraoperative AA severity in open appendectomy (*p* < 0.001), intraoperative complications (25% versus 1%, *p* < 0.001), and conversion from laparoscopic to open surgery (25% versus 0.4%, *p* < 0.001).

**Table 2:** Intraoperative data of the study groups

	Early postoperative complications (EPAC group)	No early postoperative complications (no EPAC group)	*p*-Value
	(*n* = 32)	(*n* = 553)	
Surgical approach			
Laparoscopic (*n* = 357)	14/357 (3.9%)	343/357 (96%)	0.03*
Open (228)	18/228 (7.9%)	210/228 (92.1%)	
Duration of operation in minutes (median, IQR)	74 (26)	69 (21%)	0.4
Laparoscopic grading of the severity of acute appendicitis			
Grade 0 (normal-looking appendix),	0 (0.00%)	47 (8.5%)	< 0.001*
Grade I (redness and edema)	2 (6.3%)	99 (18%)	
Grade II (fibrinous exudate),	0 (0.00%)	105 (19%)	
Grade III (segmental necrosis),	0 (0.00%)	52 (9.4%)	
Grade IV (perforation with localized appendicular abscess)	5 (15.6%)	33 (6%)	
Grade V (perforation with diffuse peritonitis)	7 (21.9%)	9 (1.6%)	
Intraoperative severity of appendicitis in the open approach			
Normally looking appendix	0 (0.00%)	23 (4.2%)	< 0.001*
Redness and edema of appendix	0 (0.00%)	45 (8.1%)	
Fibrinous exudate	0 (0.00%)	37 (6.7%)	
Localized necrosis of the appendix	4 (12.5%)	22 (4%)	
Perforation with localized appendicular abscess	2 (6.3%)	45 (8.1%)	
Perforation with diffuse peritonitis	12 (37.5%)	28 (5%)	
Intraoperative complications			
No intraoperative complications	24 (75%)	547 (99%)	< 0.001*
Urinary bladder injury	1 (3.1%)	0 (0.00%)	
Appendicular artery bleeding	0 (0.00%)	4 (0.7%)	
Omental bleeding	0 (0.00%)	2 (0.3%)	
Cecal injury	2 (6.3%)	0 (0.00%)	
Ileal injury	1 (3.1%)	0 (0.00%)	
Obscure anatomy and difficult dissection of the appendix	4 (12.5%)	0 (0.00%)	
Conversion	8 (25%)	2 (0.4%)	< 0.001*
Causes of conversion			
Urinary bladder injury repair	1 (3.1%)	0 (0.00%)	< 0.001*
Cecal injury treated with right hemicolectomy	2 (6.3%)	0 (0.00%)	
Ileal injury repair	1 (3.1%)	0 (0.00%)	
Uncontrolled bleeding from the appendicular artery	0 (0.00%)	2 (0.4%)	
Obscure anatomy and difficult dissection of the appendix	4 (12.5%)	0 (0.00%)	

*Statistically significant

The postoperative data are presented in Table 3. The most common EPAC were superficial incisional SSI (9/32,28.1%) and IAA (9/32,28.1%). Patients in the EPAC group had a significantly longer mean hospital stay (2.97 ± 1.1 versus 2.8 ± 0.93, *p* < 0.001). The mortality rate in the EPAC group was 18.8% (6/32), with causes including septic shock, respiratory failure due to COVID-19, and acute MI.

**Table 3:** Postoperative data for the study groups

	Early postoperative complications (EPAC group)	No early postoperative complications (no EPAC group)	*p*-Value
	(*n* = 32)	(*n* = 553)	
Hospital stay (days)	2.97 ± 1.1	2.8 ± 0.93	< 0.001*
Postoperative complications			
Wound infection (superficial incisional SSI)	9 (28.1%)	0 (0.00%)	< 0.001*
Wound infection(deep incisional SSI)	2 (6.3%)	0 (0.00%)	
Wound infection (organ/space infection)	2 (6.3%)	0 (0.00%)	
Intra-abdominal abscess	9 (28.1%)	0 (0.00%)	
Ileus	2 (6.3%)	0 (0.00%)	
Pneumonia	3 (9.4%)	0 (0.00%)	
Acute myocardial infarction	2 (6.3%)	0 (0.00%)	
Fecal fistula	2 (6.3%)	0 (0.00%)	
Acute adhesive intestinal obstruction	1 (3.1%)	0 (0.00%)	
Clavien–Dindo classification			
Grade 0	0 (0.00%)	553 (100%)	< 0.001*
Grade I	9 (28.1%)	0 (0.00%)	
Grade II	2 (6.3%)	0 (0.00%)	
Grade III	21 (65.6%)	0 (0.00%)	
Grade IV	0	0 (0.00%)	
Mortality	6 (18.8%)	0 (0.00%)	< 0.001*
Cause of mortality			
Septic shock	1 (3.1%)	0 (0.00%)	< 0.001*
Respiratory failure	3 (9.4%)	0 (0.00%)	
Cardiac failure	2 (6.3%)	0 (0.00%)	

*SSI* surgical site infection

*Statistically significant

Management of intraoperative and postoperative complications is summarized in Supplementary Table 2.

Multivariable logistic regression analysis showed that active COVID-19 infection (OR = 25.9; 95% CI 4.8–139.1; *p* < 0.001), ASA ≥ II (OR = 4.5; 95% CI 1.2–17.07; *p* = 0.02), surgical approach (OR = 30.6; 95% CI 8.1–115.3; *p* < 0.001), and high grade of LA ≥ IV (OR = 63.06; 95% CI, 7.5–526.4; *p* < 0.001) were risk factors associated with EPAC. Conversely, age, frailty status, Charlson Comorbidity Index, size of previous appendicular abscess, and type of drainage of the previous appendicular abscess were not associated with increased risk (Supplementary Table 4).

**Table:** Univariable and multivariable logistic regression analysis to predict Early Post-Appendectomy Complications (EPAC)

	**Univariable**			**Multivariable**	
	**OR (95% CI)**	**P-value**		**OR (95% CI)**	**P-value**
Age (years)	0.9 (0.8 - 1.03)	0.1		0.9 (0.8-1.07)	0.3
Size of Previous appendicular abscess (cm)	1.4(0.1-11.3)	0.7		-	**-**
Type of drainage of the Previous appendicular abscess	0.7(0.03-16.6)	0.8		-	**-**
Active COVID-19 infection	24.3(8.5-69. 073)	<0.001*		25.9 (4.8-139.1)	<0.001*
ASA≥II	3.5 (1.49 – 8.3)	0.004*		4.5 (1.2-17.07)	0.02*
DM	0.1 (0.063 – 0.275)	<0.001*		0.5(0.1-2.06)	0.3
BMI	1.06(0.992-1.148)	0.08		1.06(0.9-1.1)	0.1
**Frailty status**	1.2 (0.3- 4.6)	0.6			-
Charlson comorbidity index score	1.1(0.9-1.4)	0.2		1.1(0.8-1.5)	0.3
Previous abdominal surgery	1.2(0.2-5.4)	0.7		-	**-**
**Open approach**	24.3(8.5-69.07)	<0.001*		30.6(8.1-115.3)	<0.001*
Duration of operation	0.9(0.9-1.01)	0.2		0.9(.9-1.02)	0.5
Laparoscopic grading of appendicitis ≥IV	11.5(3.7-35.6)	<0.001*		63.06(7.5-526.4)	**<0.001**
Intraoperative complications	0.7(0.09-5.8)	0.7		-	-

ASA: American Society of Anesthesiologists; DM: diabetes mellitus; BMI: body mass index. *Significant P-value; OR: Odds ratio; 95% CI: 95% confidence interval

Supplementary Table 3 presents the details of EPAC. Males predominated in terms of superficial SSI, ileus, and MI, while females dominated in terms of deep/organ-space SSI. Pneumonia/MI patients were older, with higher ASA grade (III), severe frailty, and 100% mortality. Active COVID-19 infection was frequently associated with deep SSI, organ-space infection, and adhesive obstruction. Perforated appendicitis (grade IV/V) is common in intra-abdominal abscess, fecal fistula, and adhesive obstruction. The laparoscopic approach was associated with lower ileus rates.

## Discussion

This study assessed the incidence and risk factors associated with EPAC in older patients with a specific focus on the association between active COVID-19 infection and EPAC. Among 585 patients, the overall incidence of EPAC was 5.5%. The most common complications were superficial SSI, IAA, and ileus. Multivariable analysis identified ASA score ≥ II, surgical approach, active COVID-19 infection, and advanced appendicitis (grade ≥ IV) as factors statistically associated with an increased risk of EPAC.

Older patients undergoing surgery exhibit higher comorbidities, reduced physiological reserves, and altered nutritional status, resulting in increased postoperative complications and mortality rates compared with younger adults [22]. Several studies have quantified an EPAC rate between 8.3% to 63.6% [8, 9, 23–27] (Supplementary Table 4). Despite the consistently high EPAC rates reported in the literature, the present study identified a lower overall incidence of EPAC, with SSI and IAA remaining the most common. Lower complication rates might reflect differences in patient demographics, selection criteria, surgical technique (open versus laparoscopic), and stricter infection protocols adopted during the pandemic, such as mandatory mask usage and enhanced environmental sterilization protocols. Furthermore, the lower SSI rate in the LA group may be attributed to the use of endoscopic retrieval bags, which prevented direct contamination of the incision site during specimen extraction.

Nevertheless, our study showed that SSI persisted as the most frequent EPAC in older patients. This may be explained by the predominance of open appendectomy among patients with EPAC; older patients have a higher susceptibility to infections because of a lower threshold for the use of broad-spectrum antibiotics and frailty status, and carry more resistant bacterial strains [28], and secondary wound closure is not routinely employed in cases of high-grade appendicitis, potentially contributing to higher SSI rates owing to several disadvantages of secondary sutures [29]. These findings highlight the importance of enhancing infection control measures for older patients following appendectomy.

Our key finding is the strong association between active COVID-19 infection and the development of EPAC, either directly or indirectly by changing the severity of AA or the treatment approach, leading to increased SSI, higher rates of IAA, and prolonged postoperative ileus. Although causality cannot be inferred from our retrospective design, several mechanisms may underlie this association.

### Active COVID-19 infection is an associated risk factor for SSI

In our cohort, SSI rates were higher in COVID-19-positive patients, particularly after open appendectomy (OA), compared with those who underwent laparoscopic appendectomy (LA). In our study, SSI was more frequent in the OA group than in the LA group, which is consistent with previous research [30]. Although laparoscopic appendectomy (LA) is generally recommended for older patients according to guidelines, concerns regarding aerosolization of COVID-19 during pneumoperitoneum have led to a preference for open appendectomy (OA) in many centers [7]. However, we found that LA was associated with improved outcomes, including shorter hospital stays, reduced postoperative SSI, and lower all-cause mortality than OA. Given these advantages, we advocate for the continued use of LA in older patients during future pandemics, provided adequate protective measures are in place for the surgical staff.

Previous studies have reported similar trends, attributing SSI risk to COVID-19 triggers of microthrombosis and vascular injury, with elevated neutrophil extracellular traps (NETs), exacerbating tissue damage and disrupting wound repair mechanisms [31]. Additionally, active COVID-19 infection delays clinical diagnosis and, hospital bed shortages, exacerbates the severity of acute appendicitis (AA), and complicates surgical management with subsequent postoperative complications [1, 23, 32]. Our previous study confirmed that active COVID-19 infection is associated with higher-grade AA due to virus-induced appendicular artery vasculitis, which complicates surgical intervention and increases postoperative complications [33]. However, conflicting findings were reported in a previous study [34].

### Active COVID-19 infection is an associated risk factor for intra-abdominal abscesses (IAA)

IAA occurred in 28.1% of EPAC cases, with lower incidence in LA patients despite higher-grade appendicitis (grade ≥ IV). Active COVID-19 infection may increase post-appendectomy IAA risk by influencing surgical approach. While laparoscopic appendectomy (LA) is debated for complicated cases, some evidence associates CO₂ insufflation and aggressive irrigation with higher IAA rates compared with suction alone [35]. Conversely, another study reported a lower incidence of IAA following LA [36].

In the present study, IAA production rates were lower in LA than in OA. This may be attributed to controlled irrigation, early suction of pus, routine drainage in CA, and superior visualization provided by laparoscopy, which enables effective aspiration of pus from concealed spaces with irrigation of the four abdominal quadrants. Additionally, enhanced laparoscopic expertise among surgeons may improve outcomes, as suggested previously [37]. However, the role of peritoneal irrigation remains uncertain, with some studies showing no advantages over suction alone [38]. Interestingly, previous research suggests that routine abdominal drainage in CA may increase the risk of IAA, which may be due to a drain kink [39]. We believe that LA’s benefits (e.g., thorough irrigation) may decrease IAA risk, even in high-grade appendicitis, supporting its use in older COVID-19-positive patients.

### Active COVID-19 infection is an associated risk factor for post-appendectomy ileus

Ileus affected 6.3% of EPAC cases, linked to hypoalbuminemia, systemic inflammation, nutritional deficiencies, direct gastrointestinal epithelial damage via its spike protein, impair gastrointestinal motility, and OA preference during the pandemic [40–42]. Our study showed that increased use of open appendectomy (OA) during active COVID-19 is associated with a higher incidence of postoperative ileus, as reported by Yeom et al. [5]. Nutritional optimization and LA may reduce the risk of ileus in older patients with active COVID-19. Postoperative ileus in older patients with active COVID-19 infection may also reflect broader perioperative challenges, including higher ASA scores (≥ II) and systemic organ involvement. These factors complicate recovery and underscore the need for tailored anesthetic protocols (e.g., minimizing opioid use) and early mobilization strategies [43, 44]. Such measures could decrease ileus risk while addressing the multifactorial impact of COVID-19 on surgical outcomes.

## Strengths and limitations

The multicenter design of this study, incorporating both academic and nonacademic hospitals, is a major strength that improves the generalizability of the findings. However, its retrospective design introduces potential selection bias and residual confounding factors. The small number of patients in the EPAC group limits statistical power, reduces estimate precision, and restricts generalizability. The association observed between active COVID-19 infection and EPAC is likely influenced by perioperative factors, such as a higher rate of open surgery and longer operative times, and our analysis cannot establish causality. Additionally, the study did not stratify data by pandemic waves, missing changes in viral characteristics, or hospital protocols over time. Future studies addressing this gap could clarify how evolving pandemic dynamics influence the postoperative outcomes.

## Future research directions

Future research should include prospective studies using causal inference methods to determine whether the observed association between active COVID-19 and EPAC is causal. Additionally, studies should be stratified by the pandemic wave and long-term outcomes should be assessed to understand the evolving impact of active COVID-19 infection on surgical recovery in older patients.

## Conclusions

In this large, multicenter study, the incidence of EPAC was 5.5%. Active COVID-19 is significantly associated with the development of EPAC. However, the link between active COVID-19 infection and EPAC is likely confounded by coexisting risk factors, namely higher ASA scores, complicated appendicitis, and the use of open surgery. Thus, while active COVID-19 infection should be recognized as a contributing factor to perioperative risk stratification, it should not be interpreted as a direct cause. These findings highlight the need to refine surgical strategies, strengthen infection control practices, and enhance risk assessment protocols to reduce postoperative complications in older patients. Existing guidelines for managing acute appendicitis in this population should incorporate active COVID-19 infection as an associated risk factor during clinical decision-making.

## Supplementary Information

Below is the link to the electronic supplementary material.

Supplementary file1 (DOC 63 KB)

Supplementary file2 (DOC 83 KB)

Supplementary file3 (DOC 660 KB)

Supplementary file4 (DOC 100 KB)

Supplementary file5 (DOCX 38 KB)

## Data Availability

Data and material are available on request.
