# Supplementary material for: COVID-19-specific risk factor for early post-appendectomy complications (EPAC) in older patients: a retrospective study
Source: Tech Coloproctol. 2025 Nov 5;29(1):188. doi: 10.1007/s10151-025-03232-1 (PMC12589331; doi:10.1007/s10151-025-03232-1)
Supplement: Supplementary file 1 — Supplementary file1 (DOC 63 KB) [file 10151_2025_3232_MOESM1_ESM.doc]

Supplementary Table 1: Perioperative protocols and Postoperative follow-up
Surgical Approach	The diagnostic pathway and method of choice of appendectomy were LA, but the surgical approach was selected by the operating surgeon, laparoscopic equipment, expertise, and possible anaesthetic complications in line with international and national guidelines [7]	
Antibiotics	All patients received intravenous antibiotics, specifically 1 g of third-generation cephalosporins and 500 mg metronidazole, during anaesthesia induction.	
COVID-19 Precautions	Perioperative precautions during the COVID-19 pandemic were strict, as previously described [17].
The policy for assessing COVID-19 patient status involves a combination of oropharyngeal swabs analysed using real-time polymerase chain reaction (RT-PCR) and /or uncontrasted chest computed tomography (CT), with separate hospital pathways for positive patients.
	
Intraoperative Steps	LA and OA were performed as previously described [18].
·	An intraoperative swab sample was routinely performed. 
·	Initial aspiration of pus was performed, followed by controlled, non-forceful, meticulous peritoneal lavage with warm saline, which was performed gently without force until the effluent fluid appeared clear. 
·	Suture-based and Hem-O-lok closure techniques in laparoscopic appendectomy were used according to availability [19].
·	In LA, the excised appendix was removed in a specimen retrieval bag through an umbilical incision. 
·	The policy at our centre was to insert drains according to the surgeons' preference for non-complicated appendicitis and all patients with complicated AA. 
·	The incisions were closed with non-absorbable monofilament sutures immediately postoperatively. 
·	All the surgeries, including wound closure, were performed by experienced surgeons. 
·	A histological examination of the extracted appendix was performed.
	
Postoperative Care and Follow-up	·	As previously discussed, all patients followed an enhanced recovery protocol after surgery [20]. 
·	Postoperative antibiotics were not administered to the patients with uncomplicated AA. 
In complicated AA, postoperative administration of broad-spectrum antibiotics is recommended in line with the current recommendations for a minimum of 3–5 days [21]. 
·	Patients were followed up for 30 days after discharge for EPAC.	
