# Supplementary material for: COVID-19-specific risk factor for early post-appendectomy complications (EPAC) in older patients: a retrospective study
Source: Tech Coloproctol. 2025 Nov 5;29(1):188. doi: 10.1007/s10151-025-03232-1 (PMC12589331; doi:10.1007/s10151-025-03232-1)
Supplement: Supplementary file 2 — Supplementary file2 (DOC 83 KB) [file 10151_2025_3232_MOESM2_ESM.doc]

Supplementary Table 2: Dealing with intraoperative and postoperative complications.
		
	Early postoperative complications 
(EPAC group)	No early postoperative complications
(no EPAC group)	
	(n=32)	(n=553)	
Dealing with intraoperative complications	Conversion	8(25%)	2(0.4%)	
	Control of bleeding appendicular artery by laparoscopy	0(0.00%)	2(0.4%)	
	Control of bleeding omental artery by laparoscopy	0(0.00%)	2(0.4%)	
Dealing with postoperative complications	Opened wound  on bed + antibiotic	9(28.1%)	0(0.00%)	
	Reoperation and deep infection drainage	2(6.3%)	0(0.00%)	
	Reoperation and drainage of organ/space infection	2(6.3%)	0(0.00%)	
	Conservative treatment by Ryle, fluid, and electrolyte correction for ileus	2(6.3%)	0(0.00%)	
	Reoperation for fecal fistula by right hemicolectomy	2(6.3%)	0(0.00%)	
	Reoperation for acute intestinal obstruction--adhesiolysis	1(3.1%)	0(0.00%)	
	Sonar-guided aspiration for an abdominal abscess	6(18.8%)	0(0.00%)	
	Reoperation and intra-abdominal abscess drainage	3(9.4%)	0(0.00%)	
	ICU admission with cardiorespiratory support	5(15.6%)	0(0.00%)	

*statistically significant
