# Supplementary material for: COVID-19-specific risk factor for early post-appendectomy complications (EPAC) in older patients: a retrospective study
Source: Tech Coloproctol. 2025 Nov 5;29(1):188. doi: 10.1007/s10151-025-03232-1 (PMC12589331; doi:10.1007/s10151-025-03232-1)
Supplement: Supplementary file 3 — Supplementary file3 (DOC 660 KB) [file 10151_2025_3232_MOESM3_ESM.doc]

Supplementary table 3: Details of Early post-Appendectomy Complications (EPAC)
	
	wound infection(superficial incisional SSI)
(n=9)	wound infection(deep incisional SSI)
(n=2)	wound infection (organ/space infection)
(n=2)	intra-abdominal abscess
(n=9)	Ileus
(n=2)	Pneumonia
(n=3)	acute MI
(n=2)	fecal fistula
(n=2)	Acute adhesive intestinal obstruction
(n=1)	
sex	male	8(88.9%)	0(0.00%)	0(0.00%)	3(33.3%)	2(100%)	1(33.3%)	2(100%)	1(50%)	0(0.00%)	
	female	1(11.1%)	2(100%)	2(100%)	6(66.7%)	0(0.00%)	2(66.7%)	0(0.00%)	1(50%)	1(100%)	
Age(years)	65	67	70	66	65	66	74	66	69	
smoking	smoker	1(11.1%)	0(0.00%)	1(50%)	2(22.2%)	1(50%)	0(0.00%)	0(0.00%)	0(0.00%)	0(0.00%)	
	non smoker	8(88.9%)	2(100%)	1(50%)	7(77.8%)	1(50%)	3(100%)	2(100%)	2(100%)	1(100%)	
ASA	ASA-I	0(0.00%)	1(50%)	1(50%)	0(0.00%)	0(0.00%)	2(66.7%)	1(50%)	0(0.00%)	1(100%)	
	ASA-II	5(55.6%)	1(50%)	1(50%)	3(33.3%)	2(100%)	0(0.00%)	0(0.00%)	2(100%)	0(0.00%)	
	ASA-III	4(44.4%)	0(0.00%)	0(0.00%)	6(66.7%)	0(0.00%)	1(33.3%)	1(50%)	0(0.00%)	0(0.00%)	
WBCS/Cmm	16.2	15.1	15.1	16.4	16.7	15.5	16.0	15.5	14.6	
CHD( coronary heart disease)	2(22.2%)	1(50%)	1(50%)	1(11.1%)	2(100%)	0(0.00%)	1(50%)	0(0.00%)	0(0.00%)	
COVID-19 infection	Active	3(33.3%)	2(100%)	1(50%)	3(33.3%)	1(50%)	1(33.3%)	1(50%)	1(50%)	1(100%)	
	Past-history	1(11.1%)	0(0.00%)	0(0.00%)	1(11.1%)	0(0.00%)	0(0.00%)	1(50%)	0(0.00%)	0(0.00%)	
Diabetes Mellitus	Yes	8(88.9%)	0(0.00%)	0(0.00%)	7(77.8%)	1(50%)	1(33.3%)	0(0.00%)	2(100%)	0(0.00%)	
	No	1(11.1%)	2(100%)	2(100%)	2(22.2%)	1(50%)	2(66.7%)	2(100%)	0(0.00%)	1(100%)	
Body Mass Index	27	28	34	29	26	27	32	26	26	
Hypertension	hypertension	7(77.7%)	0(0.00%)	0(0.00%)	5(55.6%)	2(100%)	1(33.3%)	1(50%)	2(100%)	0(0.00%)	
	not hypertension	2(22.2%)	2(100%)	2(100%)	4(44.4%)	0(0.00%)	2(66.7%)	1(50%)	0	1(100%)	
Frailty status	managing well	2(22.2%)	0(0.00%)	0(0.00%)	0(0.00%)	0(0.00%)	0(0.00%)	0(0.00%)	0(0.00%)	0(0.00%)	
	well	1(11.1%)	0(0.00%)	0(0.00%)	1(11.1%)	0(0.00%)	0(0.00%)	0(0.00%)	0(0.00%)	0(0.00%)	
	vulnerable	1(11.1%)	0(0.00%)	0(0.00%)	1(11.1%)	1(50%)	0(0.00%)	0(0.00%)	1(50%)	0(0.00%)	
	mild frail	3(33.3%)	1(50%)	0(0.00%)	6(66.7%)	0(0.00%)	2(66.7%)	1(50%)	0(0.00%)	1(100%)	
	moderately frail	2(22.2%)	0(0.00%)	0(0.00%)	0(0.00%)	0(0.00%)	1(33.3%)	1(50%)	1(50%)	0(0.00%)	
	severely frail	0(0.00%)	1(50%)	2(100%)	1(11.1%)	1(50%)	0(0.00%)	0(0.00%)	0(0.00%)	0(0.00%)	
Charlson comorbidity  index score	4	1(11.1%)	1(50%)	0(0.00%)	0(0.00%)	0(0.00%)	0(0.00%)	0(0.00%)	0(0.00%)	0(0.00%)	
	5	6(66.6%)	1(50%)	0(0.00%)	2(22.2%)	0(0.00%)	3(100%)	1(50%)	1(50%)	0(0.00%)	
	6	0(0.00%)	0(0.00%)	0(0.00%)	3(33.3%)	0(0.00%)	0(0.00%)	1(50%)	0(0.00%)	0(0.00%)	
	7	1(11.1%)	0(0.00%)	2(100%)	1(11.1%)	1(50%)	0(0.00%)	0(0.00%)	0(0.00%)	0(0.00%)	
	8	1(11.1%)	0(0.00%)	0(0.00%)	1(11.1%)	0(0.00%)	0(0.00%)	0(0.00%)	1	0(0.00%)	
	9	0(0.00%)	0(0.00%)	0(0.00%)	1(11.1%)	1(50%)	0(0.00%)	0(0.00%)	0(0.00%)	1(100%)	
	10	0(0.00%)	0(0.00%)	0(0.00%)	0	0(0.00%)	0(0.00%)	0(0.00%)	0(0.00%)	0(0.00%)	
	11	0(0.00%)	0(0.00%)	0(0.00%)	1(11.1%)	0(0.00%)	0(0.00%)	0(0.00%)	0(0.00%)	0(0.00%)	
previous abdominal surgeries	Yes	0(0.00%)	0(0.00%)	0(0.00%)	2(22.2%)	0(0.00%)	0(0.00%)	0(0.00%)	0(0.00%)	0(0.00%)	
	No	9(100%)	2(100%)	2(100%)	7(77.8%)	2(100%)	3(100%)	2(100%)	2(100%)	1(100%)	
Previous history of episodes of acute appendicitis before the index admission	Yes	2(22.2%)	0(0.00%)	0(0.00%)	1(11.1%)	1(50%)	1(33.3%)	0(0.00%)	1(50%)	0(0.00%)	
	No 	7(77.7%)	2(100%)	2(100%)	8(88.9%)	1(50%)	2(66.7%)	2(100%)	1(50%)	1(100%)	
surgical approach	open appendectomy	5(55.6%)	1(50%)	1(50%)	6(66.7%)	2(100%)	1(33.3%)	0(0.00%)	2(100%)	0(0.00%)	
	laparoscopic appendectomy	4(44.4%)	1(50%)	1(50%)	3(33.3%)	0(0.00%)	2(66.7%)	2(100%)	0(0.00%)	1(100%)	
Duration of operation in minutes	68	73	50	72	70	79	84	83	69	
laparoscopic grading of the severity of 
acute appendicitis	Grade 0 (normal-looking appendix),	0(0.00%)	0(0.00%)	0(0.00%)	0(0.00%)	0(0.00%)	0(0.00%)	0(0.00%)	0(0.00%)	0(0.00%)	
	Grade I (redness and edema)	0(0.00%)	1(50%)	0(0.00%)	0(0.00%)	0(0.00%)	0(0.00%)	1(50%)	0(0.00%)	0(0.00%)	
	Grade II (fibrinous exudate),	0(0.00%)	0(0.00%)	0(0.00%)	0(0.00%)	0(0.00%)	0(0.00%)	0(0.00%)	0(0.00%)	0(0.00%)	
	Grade III (segmental necrosis),	0(0.00%)	0(0.00%)	0(0.00%)	0(0.00%)	0(0.00%)	0(0.00%)	0(0.00%)	0(0.00%)	0(0.00%)	
	Grade IV (perforation with localized appendicular abscess)	1(11.1%)	0(0.00%)	1(50%)	1(11.1%)	0(0.00%)	1(33.3%)	1(50%)	0(0.00%)	0(0.00%)	
	Grade V (perforation with diffuse peritonitis)	3(33.3%)	0(0.00%)	0(0.00%)	2(22.2%)	0(0.00%)	1(33.3%)	0(0.00%)	0(0.00%)	1(100%)	
Intraoperative
Severity
Of appendicitis
In
Open
approach	Normally looking appendix	0(0.00%)	0(0.00%)	0(0.00%)	0(0.00%)	0(0.00%)	0(0.00%)	0(0.00%)	0(0.00%)	0(0.00%)	
	Hyperemia of appendix	0(0.00%)	0(0.00%)	0(0.00%)	0(0.00%)	0(0.00%)	0(0.00%)	0(0.00%)	0(0.00%)	0(0.00%)	
	Fibrinous exudate	0(0.00%)	0(0.00%)	0(0.00%)	0(0.00%)	0(0.00%)	0(0.00%)	0(0.00%)	0(0.00%)	0(0.00%)	
	localized necrosis of the appendix	1(11.1%)	0(0.00%)	0(0.00%)	1(11.1%)	1(50%)	0(0.00%)	0(0.00%)	1(50%)	0(0.00%)	
	perforation with localized appendicular abscess	0(0.00%)	0(0.00%)	0(0.00%)	1(11.1%)	0(0.00%)	0(0.00%)	0(0.00%)	1(50%)	0(0.00%)	
	perforation with diffuse peritonitis	4(44.4%)	1(50%)	1(50%)	4(44.4%)	1(50%)	1(33.3%)	0(0.00%)	0(0.00%)	0(0.00%)	
Intraoperative complications	No intraoperative complications	7(77.7%)	2(100%)	2(100%)	7(77.8%)	2(100%)	2(66.7%)	1(50%)	0	1(100%)	
	Urinary bladder injury	1(11.1%)	0(0.00%)	0(0.00%)	0(0.00%)	0(0.00%)	0(0.00%)	0(0.00%)	0(0.00%)	0(0.00%)	
	Appendicular artery bleeding	0(0.00%)	0(0.00%)	0(0.00%)	0(0.00%)	0(0.00%)	0(0.00%)	0(0.00%)	0(0.00%)	0(0.00%)	
	omental bleeding	0(0.00%)	0(0.00%)	0(0.00%)	0(0.00%)	0(0.00%)	0(0.00%)	0(0.00%)	0(0.00%)	0(0.00%)	
	caecal injury	1(11.1%)	0(0.00%)	0(0.00%)	0(0.00%)	0(0.00%)	1(33.3%)	0(0.00%)	0(0.00%)	0(0.00%)	
	ileal injury	0(0.00%)	0(0.00%)	0(0.00%)	0(0.00%)	0(0.00%)	0(0.00%)	1(50%)	0(0.00%)	0(0.00%)	
	obscure anatomy and difficult dissection of the appendix	0(0.00%)	0(0.00%)	0(0.00%)	2(22.2%)	0(0.00%)	0(0.00%)	0(0.00%)	2(100%)	0(0.00%)	
Dealing with intraoperative complications			
	conversion	2(22.2%)	0	0(0.00%)	2	0	1(33.3%)	1(50%)	2(100%)	0	
	Control of bleeding appendicular artery by laparoscopy	0(0.00%)	0(0.00%)	0(0.00%)	0(0.00%)	0(0.00%)	0(0.00%)	0(0.00%)	0(0.00%)	0(0.00%)	
	Control of bleeding omental artery by laparoscopy	0(0.00%)	0(0.00%)	0(0.00%)	0(0.00%)	0(0.00%)	0(0.00%)	0(0.00%)	0(0.00%)	0(0.00%)	
Causes of conversion		
	Urinary bladder injury repair	1(11.1%)	0(0.00%)	0(0.00%)	0(0.00%)	0(0.00%)	0(0.00%)	0(0.00%)	0(0.00%)	0(0.00%)	
	caecal injury treated with right hemicolectomy	1(11.1%)	0(0.00%)	0(0.00%)	0(0.00%)	0(0.00%)	1(33.3%)	0(0.00%)	0(0.00%)	0(0.00%)	
	Ileal injury repair	0(0.00%)	0(0.00%)	0(0.00%)	0(0.00%)	0(0.00%)	0(0.00%)	1(50%)	0(0.00%)	0(0.00%)	
	uncontrolled bleeding from the appendicular artery	0(0.00%)	0(0.00%)	0(0.00%)	0(0.00%)	0(0.00%)	0(0.00%)	0(0.00%)	0(0.00%)	0(0.00%)	
	obscure anatomy and difficult dissection of the appendix	0(0.00%)	0(0.00%)	0(0.00%)	2(22.2%)	0(0.00%)	0(0.00%)	0(0.00%)	2(100%)	0(0.00%)	
hospital stay(days)	2	3(33.3%)	0(0.00%)	0(0.00%)	4(44.4%)	0(0.00%)	1(33.3%)	0(0.00%)	0(0.00%)	0(0.00%)	
	3	2(22.2%)	2(100%)	2(100%)	2(22.2%)	0	1(33.3%)	0(0.00%)	0(0.00%)	0(0.00%)	
	4	3(33.3%)	0(0.00%)	0(0.00%)	0(0.00%)	0(0.00%)	0(0.00%)	0(0.00%)	1(50%)	0(0.00%)	
	5	0(0.00%)	0(0.00%)	0(0.00%)	1(11.1%)	0(0.00%)	1(33.3%)	1(50%)	0(0.00%)	0(0.00%)	
	6	0(0.00%)	0(0.00%)	0(0.00%)	0(0.00%)	2(100%)	0(0.00%)	0(0.00%)	0(0.00%)	0(0.00%)	
	7	1(11.1%)	0(0.00%)	0(0.00%)	2(22.2%)	0(0.00%)	0(0.00%)	0(0.00%)	1(50%)	0(0.00%)	
	8	0(0.00%)	0(0.00%)	0(0.00%)	0(0.00%)	0(0.00%)	0(0.00%)	1(50%)	0(0.00%)	0(0.00%)	
	12	0(0.00%)	0(0.00%)	0(0.00%)	0(0.00%)	0(0.00%)	0(0.00%)	0(0.00%)	0(0.00%)	1(100%)	
Clavien-Dindo classification	Grade 0	0(0.00%)	0(0.00%)	0(0.00%)	0(0.00%)	0(0.00%)	0(0.00%)	0(0.00%)	0(0.00%)	0(0.00%)	
	Grade I	9(100%)	0(0.00%)	0(0.00%)	0(0.00%)	0(0.00%)	0(0.00%)	0(0.00%)	0(0.00%)	0(0.00%)	
	Grade II	0(0.00%)	0(0.00%)	0(0.00%)	0(0.00%)	2(100%)	0(0.00%)	0(0.00%)	0(0.00%)	0(0.00%)	
	Grade III	0(0.00%)	2(100%)	2(100%)	9(100%)	0(0.00%)	3(100%)	2(100%)	2(100%)	1(100%)	
	Grade IV	0(0.00%)	0(0.00%)	0(0.00%)	0(0.00%)	0(0.00%)	0(0.00%)	0(0.00%)	0(0.00%)	0(0.00%)	
Dealing with postoperative readmission		
	opened wound  on bed + antibiotic	9(100%)	0(0.00%)	0(0.00%)	0(0.00%)	0(0.00%)	0(0.00%)	0(0.00%)	0(0.00%)	0(0.00%)	
	Reoperation and drainage of deep incisional wound infection	0(0.00%)	0(0.00%)	0(0.00%)	0(0.00%)	0(0.00%)	0(0.00%)	0(0.00%)	0(0.00%)	0(0.00%)	
	conservative treatment by Ryle, fluid and electrolyte correction for ileus	0(0.00%)	0(0.00%)	0(0.00%)	00(0.00%)	2(100%)	0(0.00%)	0(0.00%)	0(0.00%)	0(0.00%)	
	reoperation for fecal fistula by right hemicolectomy	0(0.00%)	0(0.00%)	0(0.00%)	0(0.00%)	0(0.00%)	0(0.00%)	0(0.00%)	2(100%)	0(0.00%)	
	Reoperation for acute intestinal obstruction--adhesiolysis	0(0.00%)	0(0.00%)	0(0.00%)	0(0.00%)	0(0.00%)	0(0.00%)	0(0.00%)	0(0.00%)	1(100%)	
	Sonar-guided aspiration for abdominal abscess	0(0.00%)	0(0.00%)	0(0.00%)	6(66.7%)	0(0.00%)	0(0.00%)	0(0.00%)	0(0.00%)	0(0.00%)	
	reoperation and intra-abdominal abscess drainage	0(0.00%)	0(0.00%)	0(0.00%)	3(33.3%)	0(0.00%)	0(0.00%)	0(0.00%)	0(0.00%)	0(0.00%)	
	reoperation and drainage of organ/space infection	0(0.00%)	0(0.00%)	2(100%)	0(0.00%)	0(0.00%)	0(0.00%)	0(0.00%)	0(0.00%)	0(0.00%)	
	ICU admission with cardiorespiratory support	0(0.00%)	0(0.00%)	0(0.00%)	0(0.00%)	0(0.00%)	3(100%)	2(100%)	0(0.00%)	0(0.00%)	
	reoperation and deep infection drainage	0(0.00%)	2(100%)	0(0.00%)	0(0.00%)	0(0.00%)	0(0.00%)	0(0.00%)	0(0.00%)	0(0.00%)	
mortality	yes	0(0.00%)	0(0.00%)	0(0.00%)	1(11.1%)	0(0.00%)	3(100%)	2(100%)	0(0.00%)	0(0.00%)	
	no	9(100%)	2(100%)	2(100%)	8(88.9%)	2(100%)	0(0.00%)	0(0.00%)	2(100%)	1(100%)	
cause of mortality		
	septic shock	0(0.00%)	0(0.00%)	0(0.00%)	1(11.1%)	0(0.00%)	0(0.00%)	0(0.00%)	0(0.00%)	0(0.00%)	
	respiratory failure	0(0.00%)	0(0.00%)	0(0.00%)	0(0.00%)	0(0.00%)	3(100%)	0(0.00%)	0(0.00%)	0(0.00%)	
	cardiac failure	0(0.00%)	0(0.00%)	0(0.00%)	0(0.00%)	0(0.00%)	0(0.00%)	2(100%)	0(0.00%)	0(0.00%)	
