# Supplementary material for: COVID-19-specific risk factor for early post-appendectomy complications (EPAC) in older patients: a retrospective study
Source: Tech Coloproctol. 2025 Nov 5;29(1):188. doi: 10.1007/s10151-025-03232-1 (PMC12589331; doi:10.1007/s10151-025-03232-1)
Supplement: Supplementary file 4 — Supplementary file4 (DOC 100 KB) [file 10151_2025_3232_MOESM4_ESM.doc]

study	Patient criteria (number, age)	Surgical approach	Overall EPAC rate	Most frequent EPAC	Other rates and mortality	
Angeramo et al. [8].	122 , ≥65 years	LA	30%	IAA to be the most common complication (7%)	no reported deaths	
Weinandt et al. [9]	2060, ≥75 years	LA,OA	46%	superficial SSI occurred in 15.4% of cases, IAA in 4.6%, pulmonary complications and septicemia each in 1.5%	30-d mortality was 6.2% for elderly patients≥75 years	
Segev et al. [23].	68, ≥68 years	Laparoscopic appendectomy (LA),open appendectomy (OA)	34.3%	superficial surgical site infection (SSI) (9%)and intra-abdominal abscess (IAA)(1.5%)	Only one patient (aged 82 years) died in the early postoperative period	
McGowan et al. [24].	33, > 80 years old	OA(90%),LA	63.6%	post-operative ileus (n=7; 21%), SSI (n = 5; 15.2%) and pneumonia (n = 5; 15.2%)	Not recorded	
Hui et al. [25].	95, ≥70 years	LA,OA	37.5%	SSI, IAA, ileus, arrhythmia, pneumonia, urinary retention, sepsis, and acute renal failure	mortalities occurred in 3 patients and attributed to sepsis and cardiopulmonary arrest	
Stevens et al. [26].	NSQIP Cohort: 349,909 patients (51.1% male, median age 36 years, IQR 26–51; 8.4% aged ≥65).
VASQIP Cohort: 14,619 patients (89.1% male, median age 51 years, IQR 35–63; 21% aged ≥65).
	laparoscopic appendectomy (LA)	NSQIP Cohort:
9.8% at age 65,11.9% at age 75, and 14.5% at age 85 
VASQIP Cohort:
7.5% at age 65 ,8.3% at age 75 ,and 9.1% at age 85	SSI, pneumonia, sepsis/septic shock, renal failure, myocardial infarction (MI), pulmonary embolism.	NSQIP Cohort:
0.12% at age 65, 1.7% at age 85 
VASQIP Cohort: 0.38% at age 65 , 2.17% at age 85 	
Kwon et al. [27]. 
	160 (132 aged 65–79 years and 28 aged ≥80 years)	LA,OA	Total Complications: 24.4% (39/160).
≥80 group: 32.1% (9/28).
65–79 group: 22.7% (30/132).
	SSI (12.9% vs. 10.7%), ileus (6.1% vs. 7.1%), and pulmonary complications (2.3% vs. 14.3%) 	no reported deaths	
Supplementary Table 4: studies quantified Early post-appendectomy complications (EPAC)
